# Near-infrared light-triggered nano-prodrug for cancer gas therapy

**DOI:** 10.1186/s12951-021-01078-x

**Published:** 2021-12-23

**Authors:** Runcong Liu, Yongjun Peng, Ligong Lu, Shaojun Peng, Tianfeng Chen, Meixiao Zhan

**Affiliations:** 1 Zhuhai Precision Medical Center, Guangdong Provincial Key Laboratory of Tumor Interventional Diagnosis and Treatment, Zhuhai Hospital Affiliated With Jinan University (Zhuhai People’s Hospital), Jinan University, Zhuhai, 519000 Guangdong P.R. China; 2 College of Chemistry and Materials Science, Guangdong Provincial Key Laboratory of Functional Supramolecular Coordination Materials and Applications, Jinan University, Guangzhou, 510632 China

## Abstract

Gas therapy (GT) has attracted increasing attention in recent years as a new cancer treatment method with favorable therapeutic efficacy and reduced side effects. Several gas molecules, such as nitric oxide (NO), carbon monoxide (CO), hydrogen (H_2_), hydrogen sulfide (H_2_S) and sulfur dioxide (SO_2_), have been employed to treat cancers by directly killing tumor cells, enhancing drug accumulation in tumors or sensitizing tumor cells to chemotherapy, photodynamic therapy or radiotherapy. Despite the great progress of gas therapy, most gas molecules are prone to nonspecific distribution when administered systemically, resulting in strong toxicity to normal tissues. Therefore, how to deliver and release gas molecules to targeted tissues on demand is the main issue to be considered before clinical applications of gas therapy. As a specific and noninvasive stimulus with deep penetration, near-infrared (NIR) light has been widely used to trigger the cleavage and release of gas from nano-prodrugs via photothermal or photodynamic effects, achieving the on-demand release of gas molecules with high controllability. In this review, we will summarize the recent progress in cancer gas therapy triggered by NIR light. Furthermore, the prospects and challenges in this field are presented, with the hope for ongoing development.

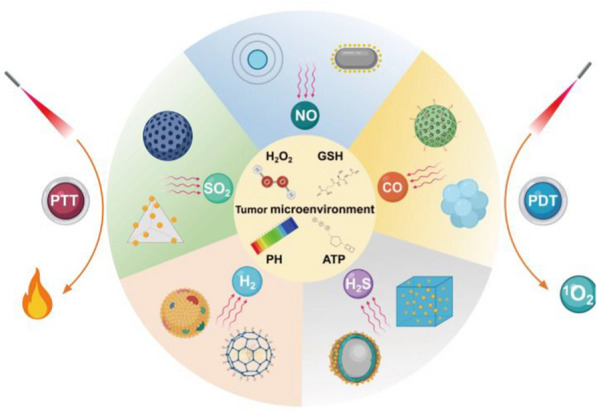

## Introduction

**Scheme 1:**
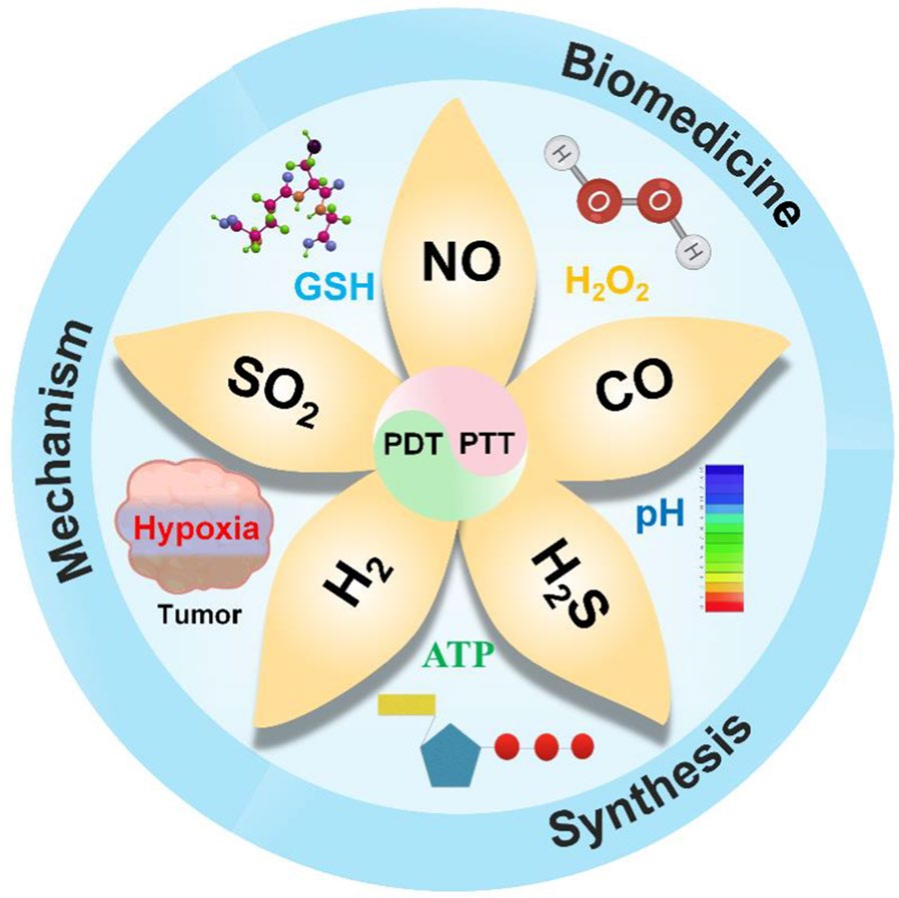
Schematic illustration of NIR light triggered nano-prodrug for cancer gas therapy

Cancer is one of the most serious diseases that threaten human health worldwide [1–5]. At present, cancer treatments mainly include surgery, chemotherapy and radiotherapy, but the clinical benefits are unsatisfactory owing to the heterogeneity and complexity of cancer [6–9]. In recent years, as a new cancer treatment model, gas therapy has played important roles in cancer treatment with high therapeutic efficiency [10–14]. The commonly used therapeutic gas molecules include nitric oxide (NO), carbon monoxide (CO), hydrogen (H_2_), hydrogen sulfide (H_2_S), and sulfur dioxide (SO_2_) (Scheme 1). The development of the first biomedical gas, NO, led to the Nobel Prize in Physiology and Medicine in 1998 for its significant therapeutic effects on cardiovascular diseases [15]. Subsequently, other therapeutic gas molecules have also been used in biomedical applications, especially cancer treatment. Although gas therapy has made great progress in the treatment of diseases, most therapeutic gases are prone to nonspecific distribution after systemic administration, resulting in strong irritation to the respiratory system and severe side effects on normal tissues [10, 16]. Furthermore, the off-targeting phenomenon of gas molecules often leads to inferior tumor accumulation and weakens therapeutic efficiency [17]. Therefore, the development of stimulus-responsive gas-releasing nanoplatforms (GRNs) for controlled gas release is the main goal that needs to be achieved before the clinical application of gas therapy [18, 19]. Stimulus-responsive GRNs could effectively prevent gas from being released prematurely in blood circulation or in normal tissues, preventing possible toxicity and side effects [20, 21]. Furthermore, stimulus-responsive GRNs could target tumor tissues and release gas molecules in a controlled manner, significantly enhancing the antitumor effect. There are two main approaches to stimulating the release of gas molecules: (1) endogenous stimuli, such as weak acidity and high H_2_O_2_, glutathione (GSH), ATPase (ATP) and special enzyme levels; and (2) exogenous stimuli, such as light, sound, electricity and magnetism [22–27]. The response to internal stimuli can realize the controlled release of gas without external stimulation, which is simple and convenient without damage to normal tissues [28–31]. In contrast, an external stimulus source has the advantage of easy control of the stimulus source to accurately control the gas release rate, quantity and tissue retention [22, 23, 32]. Among exogenous stimuli, lasers have the advantages of convenience and effectiveness, leading to the wide application of photocontrolled GRNs [33–37]. Compared to ultraviolet or visible light with limited tissue penetration depth, NIR light has higher tissue penetration depth and lower phototoxicity and therefore exhibits broader application prospects in cancer gas treatment [38–40].

NIR can not only trigger the controlled release of gas molecules from GRNs but also achieve phototherapy, including photothermal therapy (PTT) and photodynamic therapy (PDT), for combined antitumor efficacy [41]. PTT uses a photosensitizer to convert absorbed light energy into thermal energy, which produces local high temperatures to destroy tumor cells [42–50]. PTT has the advantages of low invasiveness, deep tissue penetration, high spatiotemporal precision and low cytotoxicity [51–57]. Compared to PTT, PDT has a more complex basic mechanism. PDT can induce the apoptosis of cancer cells by stimulating the production of reactive oxygen species (ROS), such as hydroxyl radicals, singlet oxygen and superoxide dismutase, after irradiation with ultraviolet or NIR light [58–63]. ROS can also increase the permeability of the tumor cell membrane and enhance nanoparticle uptake, a phenomenon known as photochemical internalization [64–67]. The precise release of gas molecules from GRNs in diseased tissue is a prerequisite to ensure the effectiveness and biosafety of gas therapy [18, 19]. At present, many kinds of GRNs have been designed to transport gas to tumor tissues, such as poly(D-L-lactic-co-glycolic acid) (PLGA), micelles, silica/mesoporous silica, organosilica, MnO_2_, graphene, Bi_2_Se_3_, upconversion nanoparticles (UCNPs), and CaCO_3_[20]. In addition, some GRNs can be loaded with antineoplastic drugs that can accumulate in the tumor through the enhanced permeability and retention (EPR) effect or active targeting and regulate relevant proteins to reverse the multidrug resistance (MDR) of tumor cells, thus enhancing the antitumor effects of chemotherapeutic drugs [68–70]. Due to the diversity of therapeutic gases and treatment mechanisms, GRNs could achieve efficient therapeutic effects and reduce side effects at the same time [71, 72]. Table 1 summarizes the applications of various representative NIR-responsive gas prodrugs in tumors in recent years.

**Table 1:** Representative NIR-responsive nano-gas prodrugs and their biomedical applications

Gas	Materials	Tumor cells lines	Therapy	References
NO	RBS-UCNPs GO-BNN_6_ Me-RBSs Nb_2_C–MSNs–SNO BNN-Bi_2_S_3_ Fe_3_O_4_@PDA@Ru-NO@FA PTNGs PNOC-PDA/DOX DTX@m-PB-NO SPNs PFTDPP Lyso-Ru-NO@FA@C-TiO_2_ L-Arg@PCn@Mem DPP-NF _AD_Au@CuS YSNPs	Human breast cancer MCF-7/DOXR cells Human osteosarcoma 143B cells Human breast cancer 4T1-LUC cells Mouse breast cancer 4T1 cells Human liver cancer BEL-7402 cells Human cervical carcinoma HeLa cells Human breast cancer MCF-7/ADR cells Human breast cancer MCF-7/ADR cells Mouse breast cancer 4T1 cells Human breast cancer MCF-7 cells Human breast cancer MCF-7 cells Mouse breast cancer 4T1 cells Human cervical cancer HeLa cells Human breast cancer MCF-7/ADR cells	PTT PTT PTT PTT PTT PTT PTT PTT PTT PTT PDT PDT PDT PDT	[73] [74] [75] [76] [77] [78] [79] [80] [81] [82] [83] [84] [85] [86]
CO	m-PB-CO PdNS-CO POM-anchored HMON FeCO-DOX@MC MCM@PEG-CO-DOX PEG@DW/BC CORM@G_3_DSP-CE_6_ Uio-BDP-MnCO PB-CO-TPZ PPPPB − CO − Dox NPs NCu-FleCP Fe(CO)_5_@Au	Human cervical cancer HeLa cells Human lung cancer A549 cells Human malignant glioma U87MG cells Mouse breast cancer 4T1 cells Human colon tumor HCT116 cells Mouse colorectal cancer CT26 cells Mouse breast cancer 4T1 cells Human breast cancer MCF-7 cells Mouse breast cancer 4T1 cells Human breast cancer MCF-7/ADR cells Mouse breast cancer 4T1 cells Mouse breast cancer 4T1 cells	PTT PTT PTT PTT PTT PTT PTT PTT PDT PDT PDT PDT	[87] [88] [89] [90] [91] [92] [93] [94] [95] [96] [97] [98]
H_2_S	SP-loaded PEG-UCNPs rGO–PEI-DTC AB-DS@BSA-N_3_ ZnS@ZIF-8	Human breast cancer MCF-7 cells Human breast cancer MCF-7 cells Human laryngeal cancer Hep2 cells Human hepatocellular carcinoma Huh7 cells	PTT PTT PTT PDT	[99] [100] [101] [102]
H_2_	PdH-MOF mPDAB NPs Z-scheme SnS1.68–WO2.41 UCCZ NPs	Human cervical cancer HeLa cells Mouse breast cancer 4T1 cells Mouse breast cancer 4T1 cells Mouse breast cancer 4T1 cells	PTT PTT PTT PDT	[103] [104] [105] [106]
SO_2_	DNs–Naph–Cbl RUCSNs-DM Au-Ag-BTS HTNs GNRS@PDA-BTS	Human breast cancer MDA-MB-231 cells Human colon cancer S180 cell Mouse breast cancer 4T1 cells Human breast cancer MCF-7 cells	PTT PDT PDT PDT	[107] [108] [109] [110]

In this review, the latest progress in gas prodrugs excited by NIR lasers in tumor therapy is systematically reviewed. First, the reasonable design of multifunctional GRNs is introduced. Second, the advantages of GRNs, including five gas molecules (NO, CO, H_2_, H_2_S, SO_2_), in tumor therapy under NIR laser irradiation are summarized. Then, combinations of gas therapy with PTT and PDT are introduced, which reveals the synergistic antitumor mechanism of GRNs. Finally, the challenges and possible solutions of gas therapy under NIR laser radiation are discussed.

## NIR light-triggered NO prodrug

NO was the first gas signaling molecule used and was found to play important roles in a series of physiological processes, such as apoptosis, angiogenesis, and immune response [111–116]. In addition NO can deplete collagen through inducing matrix metalloproteinases (MMP), which has been used for improved nanoparticle penetration in solid tumors [117, 118]. The physiological function and clinical application of NO have a longer research history than those of other gas molecules [119]. NO plays a dual role in tumor therapy: it can promote cancer growth at low concentrations (< 1 μM) but exhibits antitumor effects at high concentrations (> 1 μM) [120–122]. Therefore, tumor growth can be effectively inhibited by raising the concentration of NO above the basic level in tumor tissues. In addition, NO can enhance the therapeutic effects of chemotherapeutic drugs by overcoming the MDR of cancer cells, and it can improve the efficacy of PDT by reacting with ROS to form highly toxic peroxynitrites [84]. In exogenous stimulation wiht NIR laser irradiation, the position, duration and dose of the light source can be accurately controlled, offering practical application value in biomedical use.

### Photothermal therapy-triggered NO prodrug

**Fig. 1:**
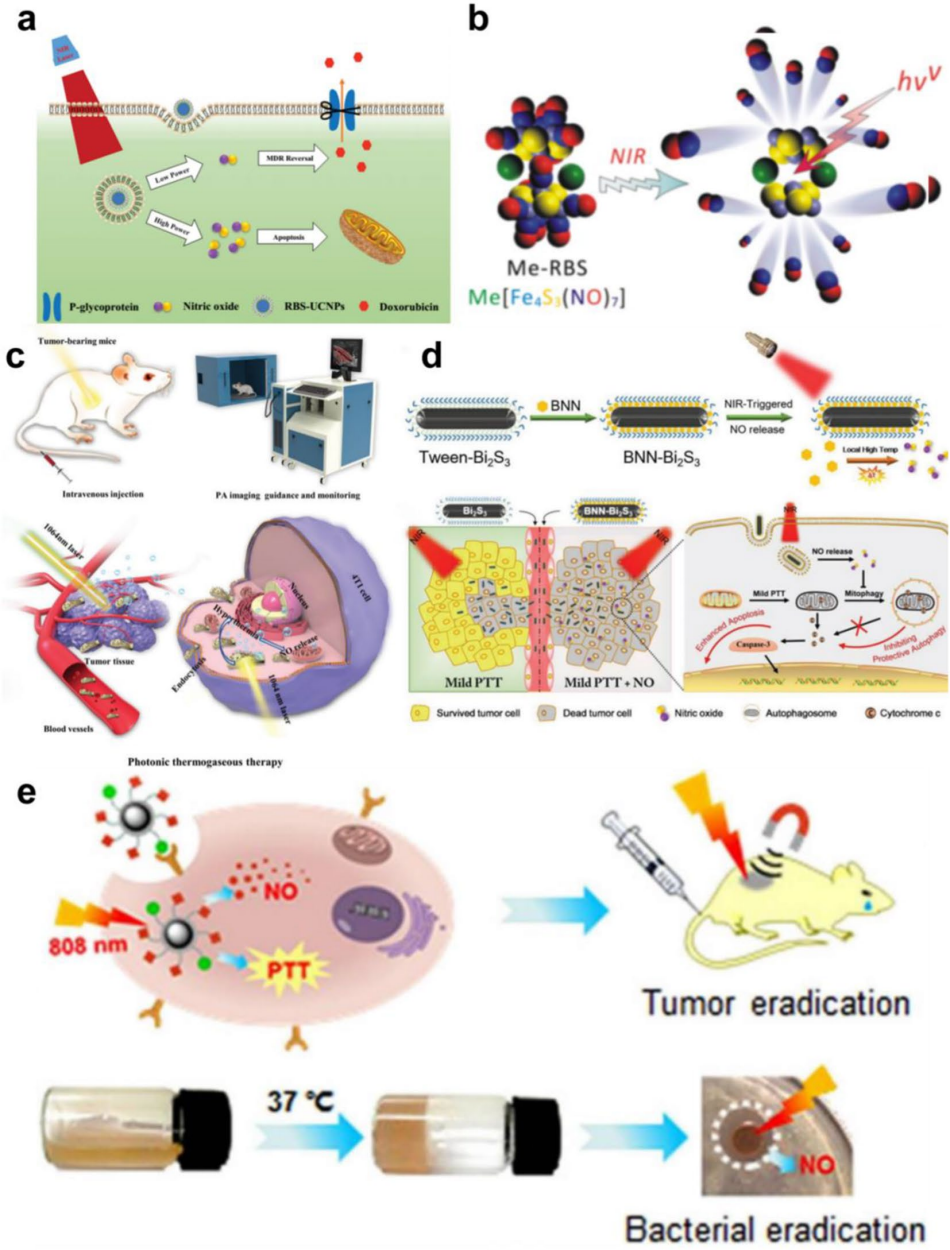
**a** Schematic illustration of 980-nm laser light-triggered on-demand NO release for dose-dependent therapeutic applications. Reproduced with permission from Ref [73]. Copyright 2015, Wiley–VCH. **b** Schematic illustration of the coordination-precipitation process of Me-RBS and NIR-responsive release of NO. Reproduced with permission from Ref [75]. Copyright 2017, American Chemical Society. **c** Schematic illustration of theranostic functions of Nb_2_C–MSNs–SNO, including free delivery within blood vessel after intravenous injection, photothermal-triggered NO release, photonic thermogaseous therapy toward oncotherapy, and PAI guidance and monitoring. Reproduced with permission from Ref [76]. Copyright 2019, Wiley–VCH. **d** Schematic illustration of synthetic procedure and NIR-triggered NO release property of BNN-Bi_2_S_3_, and synergistic mechanism of NO and mild PTT in cancer therapy. Reproduced with permission from Ref [77]. Copyright 2019, Wiley–VCH. **e** Schematic of the nanoplatform (1) for target-directed delivery of NO and production of PTT under 808 nm light irradiation, and schematic of the preparation of the CS-PVA/NO hydrogel and its antibacterial effect by NO under irradiation with 808 nm light. Reproduced with permission from Ref [78]. Copyright 2020, American Chemical Society

Photothermal therapy is the use of NIR light radiation lesions of organic or inorganic nanomaterials to generate local heat in the tumor, stimulating the release of NO in gas therapy [123, 124]. At present, various NO prodrugs have been developed, including organic nitrates/nitrites, metal-NO complexes, nitrosamines, and S-nitrosomercaptan. Moreover, the photothermal conversion and thermosensitive properties give these NO nano-prodrugs the ability to absorb NIR and convert it into heat, thus promoting the breaking of chemical bonds to release NO [125]. Therefore, various methods to release NO by using the photothermal effect produced by NIR have been developed in recent years. For example, Zhao et al. reported an NIR-triggered NO release platform based on UCNPs and photosensitive ruxin black salts (RBS-UCNPs), which can capture 980 nm NIR photons and convert them into higher-energy Ultraviolet–visible (UV–vis) photons. In addition, the white upconversion emission causes the maximum spectrum to overlap with the absorption peak of RBS, and then the effective photolysis of NO is induced by the energy transfer (ET) process under irradiation with a 980 nm laser. This work proved that a high concentration of NO produced by high-intensity NIR can directly kill cancer cells, while a low concentration of NO can overcome MDR in chemotherapy by inhibiting the expression of P-glycoprotein (P-gp) on the cancer cell membrane (Fig. 1a) [73]. Although the nanodrugs designed by Zhao et al. have a good NIR response and significant antitumor MDR, they have a low drug loading rate and poor light transmission efficiency (NIR-UV light). High-power NIR laser irradiation is usually required to produce enough NO, which inevitably leads to potential thermal damage. To solve this problem, Chen et al. constructed a novel sandwich nanodrug (GO-BNN6) with NIR light response by π-π stacking of graphene oxide (GO) nanoparticles with an NO donor (BNN6) that has high drug loading and thermal stability. GO can absorb NIR photons of 808 nm into active electrons, inducing BNN6 decomposition to release NO, which has significant anticancer effects. Importantly, GO-BNN6 nanopharmaceuticals have repeatable NIR-controlled NO release and high sensitivity to NIR radiation power density, which helps to achieve accurate on-demand release of NO while reducing the risk of NO poisoning [74].

According to previous studies, the antitumor effect of NO is closely related to its concentration [126]. However, NO molecules have a short half-life under physiological conditions and are easily consumed by free radicals or biological macromolecules [127]. Therefore, it is difficult to achieve effective concentrations of NO in the tumor area [126, 127]. In another study, Qian et al. developed a fast, simple and efficient coordination precipitation route for insoluble metal ruxin black salts (MeRBs), which have a stable photoresponse, low cytotoxicity and high thermal stability. Under 808 nm laser stimulation, Me-RBS can absorb light energy and stimulate the release of NO as needed. However, when the NIR light irradiation was stopped, NO release stopped almost completely. This confirms that the release of NO by Cu-RBS has high NIR controllability. In addition, in the mouse 4T1-Luc breast cancer model, compared with the blank control group, the NIR irradiation alone group and injection of Cu-RBS alone group had no significant inhibitory effect on the growth of primary 4T1-Luc tumors in mice. However, the combined injection of Cu-RBS and NIR light significantly inhibited the growth of primary 4T1-Luc tumors in. Bioluminescence imaging and Masson and H&E staining analyses of pulmonary metastasis clearly showed proliferative cancer nodules in the three control groups (PBS, NIR, Cu-RBS), but no obvious cancer nodules were seen in the Cu-RBS + NIR group. In brief, Cu-RBS, as a NO donor stimulated by NIR, can effectively inhibit the growth and metastasis of metastatic breast cancer (Fig. 1b) [75]. Recently, Xu et al. proposed their own design of an NO release nanoreactor based on an Nb_2_CMXene nanosheet and a NO donor (S-nitrosomercaptan, RSNO). Compared to other NO donors, RSNO has the unique advantage of high biocompatibility. Under the irradiation of 1064 nm laser, MXene can produce heat shock, which triggers the breakage of S-NO bond in RSNO to release NO precisely. This effectively increases the concentration of NO in the tumor area and further induces apoptosis.In addition, NB2C-MSNS-SNO has excellent PA imaging effect. With the increase of concentration and time, the contrast of PA imaging becomes higher, which can be used as an excellent PA contrast agent.At the same time, the nanomedicine has good biocompatibility and can be quickly eliminated from the body by the kidney, which has great potential for clinical transformation (Fig. 1c) [76].

In addition, Zhao et al. combined bismuth sulfide (Bi_2_S_3_) nanoparticles as carriers with the NO donor bis-N-nitroso compound (BNN) to construct efficient NIR-triggered NO-releasing nanocomposites. Bi_2_S_3_ can be targeted to tumor tissue, converting the light energy of the 1064 nm laser into thermal energy to trigger BNN decomposition and the release of NO. At the same time, NO can maintain the expression of p62 gene to inhibit protective autophagy, which could enhance the efficacy of PTT by aggravating thermal injury (Fig. 1d) [77]. Among many NO donors, ruthenium nitrite (Ru-NO) not only has good biocompatibility and low cytotoxicity under physiological conditions but also releases NO controllably under NIR light irradiation [128, 129]. Therefore, Liu et al. covalently linked a Ru-NO donor to the Folic Acid (FA) targeting group on the Fe_3_O_4_@PDA magnetic carrier to construct a new multifunctional magnetic nanoplatform that can produce an obvious photothermal effect under 808 nm laser irradiation, which stimulates the release of NO. Moreover, the nanoplatform can target and be transported to tumor cells under the guidance of a magnetic field and FA targeting groups, which has obvious antitumor effects (Fig. 1e) [78].

**Fig. 2:**
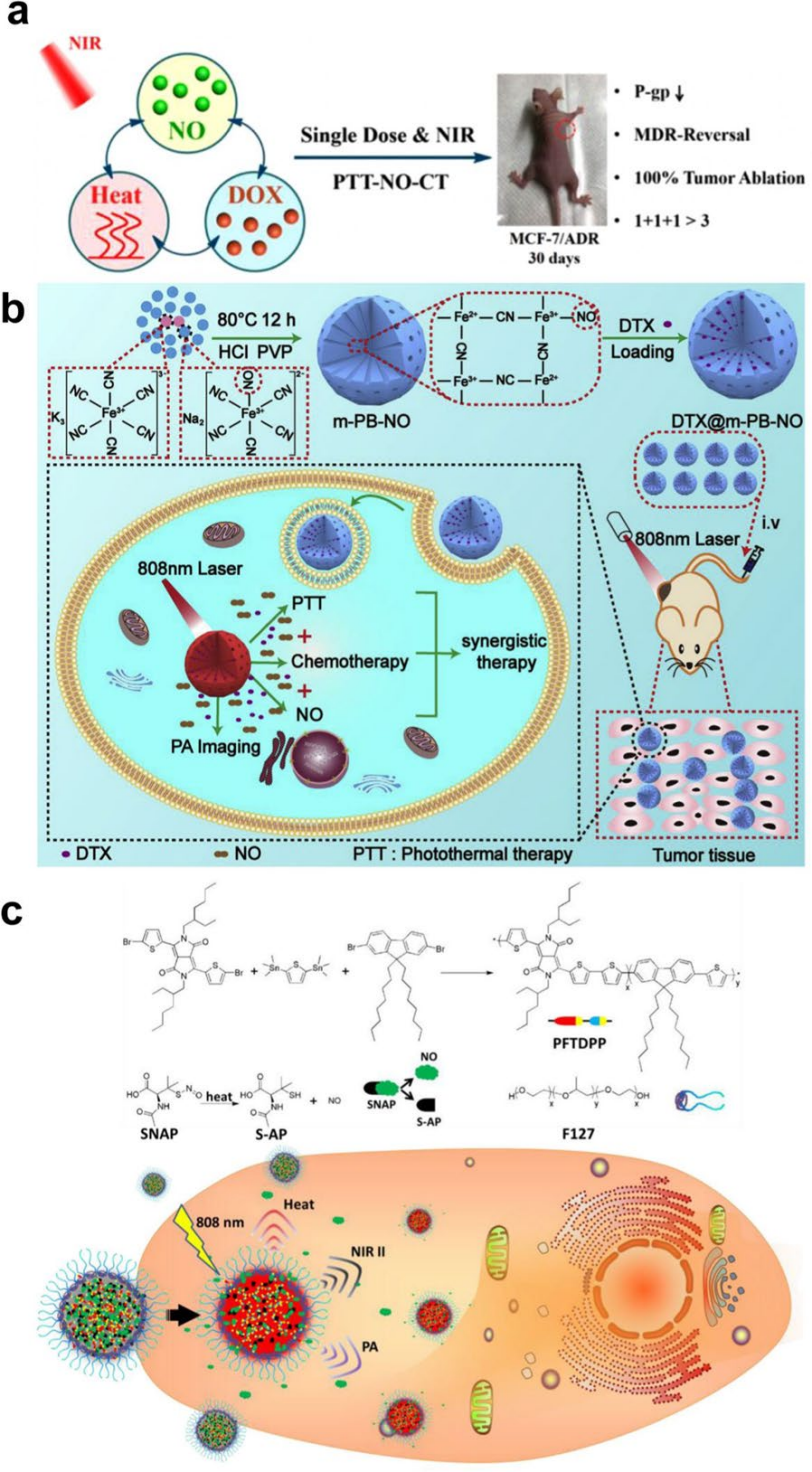
**a** Therapeutic effect of NIR light guided triple therapy on MCF-7/ADR in vitro. Reproduced with permission from Ref [80]. Copyright 2019, American Chemical Society. **b** Schematic illustration of the preparation process and in vivo behavior of m-PB-NO. Reproduced with permission from Ref [81]. Copyright 2019, American Chemical Society. **c** NIR-II/photoacoustic imaging-guided photothermal initiated NO/photothermal therapy. Reproduced with permission from Ref [82].Copyright 2019, American Chemical Society

The MDR of tumor cells is one of the main obstacles leading to failure of tumor chemotherapy [130]. According to recent studies, overexpression of P-glycoprotein (P-gp) in tumor cells can cause chemotherapeutic drugs (such as doxorubicin and paclitaxel) to be pumped out of the cells [131]. However, NIR stimulation-responsive polymer nanoparticles (such as drug-loaded or drug-bound nanoparticles) can overcome MDR by inhibiting the expression of P-gp. Therefore, Yang et al. developed a Fe_3_O_4_@polydopamine photothermal platform with high drug loading, which was directly triggered by 808 nm laser irradiation to efficiently release NO and reduce the expression of the P-gp protein, overcoming the MDR of tumor cells during chemotherapy [79]. At present, several polypeptide-polyoxyethylene drug-loaded micelles have been approved by the Food and Drug Administration (FDA) to enter clinical antitumor trials [132]. Therefore, Dong et al. designed an NIR-responsive drug-loaded peptide nanocomposite (PNOC-PDA/DOX) by coupling poly(L-cysteine)_20_-poly(ethylene oxide)_45_ (PC) with S-nitroso(SNO) and embedding biomimetic dopamine (PDA) and DOX. Under 808 nm laser irradiation, PTT can induce the pyrolysis of S-NO to release NO. PNOC-PDA/DOX exhibits pH-responsive drug release. In the acidic tumor microenvironment, protonation of the amino group on DOX destroys π − π stacking to trigger drug release. NO can also overcome the MDR of tumors, enhancing their chemosensitivity by significantly inhibiting the expression of P-gp (Fig. 2a) [80].

Sodium nitroprusside (SNP) is not only a commonly used drug in the treatment of hypertension but also an NO donor [74, 75]. Zhang et al. synthesized hollow mesoporous Prussian blue loaded with docetaxel (DTX@m-PB-NO) using SNPs. Under 808 nm laser irradiation, DTX@m-PB-NO can realize triple therapy with NO gas therapy, PTT and chemotherapy at the same time. In a mouse tumor model, DTX@m-PB-NO not only showed an obvious ability to kill tumors under NIR irradiation but also inhibited tumor lung metastasis. Picric acid staining was used to detect lung metastatic nodules. There were almost no lung metastatic nodules in the DTX@m-PB-NO + NIR group, while obvious lung metastatic nodules could be seen in the control groups (Fig. 2b) [81]. Recently, Fan et al. constructed semiconductor polymer nanoparticles (SPNs PFTDPP) by using the S-nitrosomercaptan group (SNAP) as a NO donor. Under the thermal energy produced by 808 nm laser radiation, SNAP can undergo thermal decomposition into NO. In addition, PFTDPP exhibits an obvious fluorescence signal in the NIR II region under808 nm laser irradiation, enabling fluorescence imaging. Therefore, PFTDPP SPNs can use fluorescence imaging characteristics to guide NO gas therapy and PTT (Fig. 2c) [82].

### Photodynamic therapy-triggered NO prodrug

**Fig. 3:**
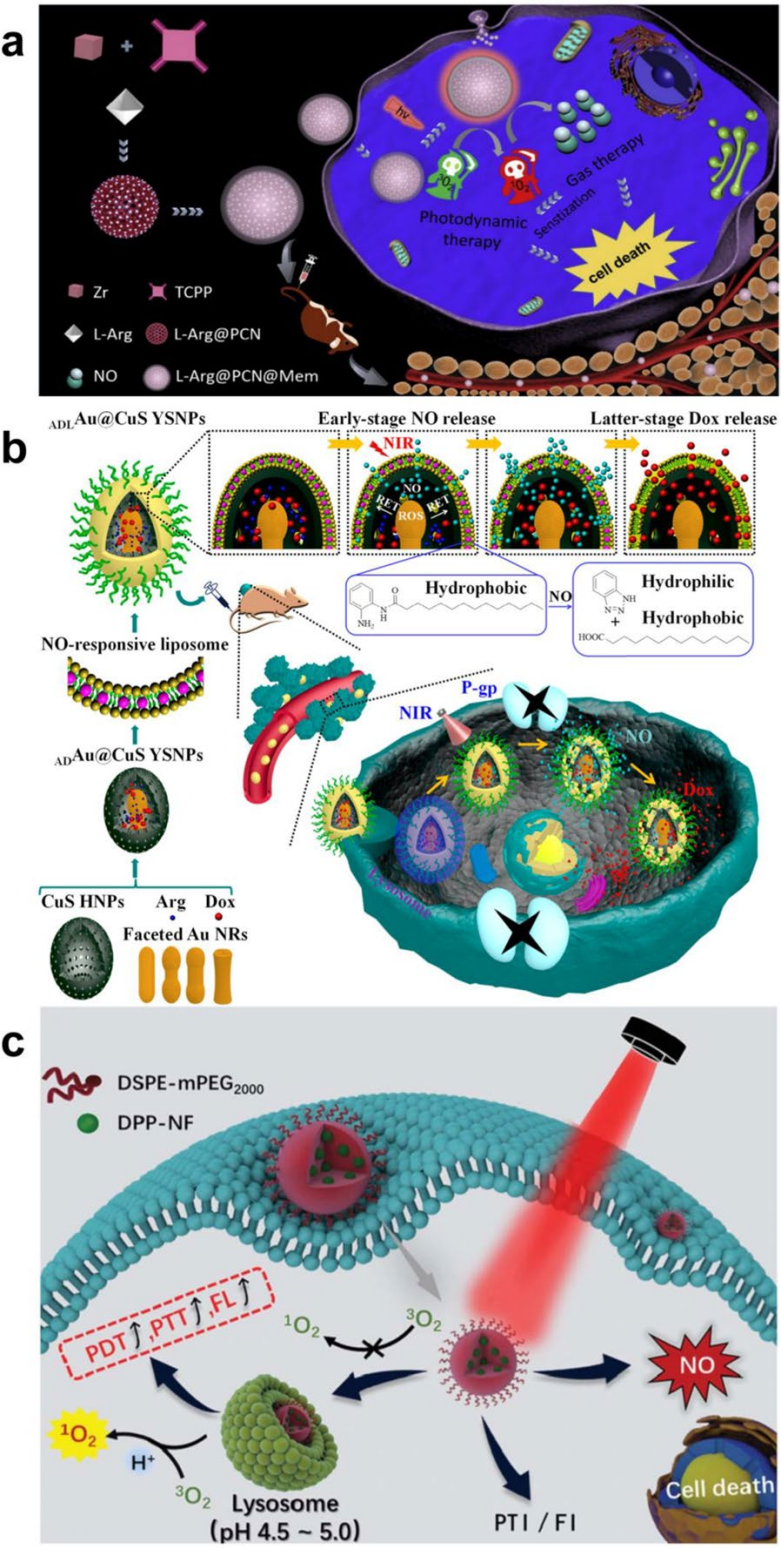
**a** Schematic illustration of L-Arg@PCN@Mem preparation and lethal mechanism of gas therapy and sensitized photodynamic therapy against tumor cells. Reproduced with permission from Ref [84]. Copyright 2018, Elsevier Ltd. **b** Illustrating the NO and Dox programmable release and MDR cancer therapy of _ADL_Au_2_@CuS YSNPs. Reproduced with permission from Ref [85]. Copyright 2018, American Chemical Society. **c** Schematic illustration of pH-responsive DPP-NF NPs for PTI and FI guided PDT/PTT/GT synergistic cancer therapy. Reproduced with permission from Ref [86]. Copyright 2019, The Royal Society of Chemistry

In antineoplastic therapy, PDT can also be used to trigger the controlled release of NO, which could inhibit the expression of P-gp protein and improve the sensitivity of cancer cells to chemotherapeutic drugs [133, 134]. In addition, NO can directly or indirectly react with ROS to form highly active peroxynitrite (ONOO^−^) molecules, improving the efficacy of gas therapy and PDT [133, 135]. According to many recent studies, lysosomes are closely related to the programmed death of cancer cells [136]. Therefore, Liu et al. developed a novel multifunctional NO delivery platform for cancer cell lysosome targeting. The nanoplatform can selectively target cancer cells overexpressing folate receptor (FR) and enrich in the lysosomes of cancer cells. Under 808 nm laser irradiation, nanoparticles can undergo phototriggered electron and energy transfer in lysosomes. This process will stimulate the release of NO and ROS from the NO donor and surrounding O_2_, resulting in lysosome damage and promoting the programmed death of cancer cells [83]. However, once the nanoparticles enter the human body, they will be easily cleaned by the human immune system and captured by capillaries, which would decrease the drug concentration at the tumor site, reducing the therapeutic effects [137]. To overcome this problem, Zhang et al. prepared a porous coordination network (PCN) containing the NO donor L-arginine (L-Arg) inside a cancer cell membrane. The biomimetic multifunctional nanosystem (L-Arg@PCn@Mem) exhibits good homologous targeting to tumor tissues, which can avoid immune cleaning and take advantage of EPR at the tumor site. In addition, once PCN is irradiated by a 660 nm laser, it will produce a large number of free radicals, which can convert L-Arg into NO. The ROS-stimulated production of NO can enhance the effect of PDT under hypoxia to realize combined gas therapy and PDT (Fig. 3a) [84].

Diketopyrrolopyrrole (DPP) derivatives are efficient photosensitizers that have the advantages of NIR absorption, high light stability and thermal stability [138–140]. Recently, Dong et al. designed pH-sensitive DPP nanoparticles (DPPNF) loaded with NO photodonors (4-nitro-3-trifluoromethylaniline, NF) and pH-sensitive groups (dimethylaminophenyl). DPPNF can be activated in the acidic environment of lysosomes, enhancing the ROS production and photothermal effects. Under 660 nm NIR light irradiation, NF can achieve the controllable release of NO under light/dark conditions, which induces lysosomal damage to enhance the efficacy of PDT, leading to tumor cell apoptosis. It shows excellent tumor lethality in gas therapy, PTT and PDT (Fig. 3b) [85]. In another work, Zhang et al. developed a complex liposome nanosystem that can sequentially release NO and DOX. Under 808 nm laser irradiation, nanoparticles with Au-NRs as the core undergo resonance energy transfer (RET) to produce a large amount of ROS. L-Arg can be converted to NO by NO synthetase with ROS-induced activity. When liposomes embedded with hydrophobic o-phenylenediamine lipids encounter NO gas molecules, the o-phenylenediamine lipids can change from hydrophobic to hydrophilic. This process destroys the phospholipid bilayer of liposomes and eventually releases DOX. At the same time, NO can inhibit the expression of P-gp, creating a favorable microenvironment for the later release of DOX accumulation (Fig. 3c) [86].

## NIR light-triggered CO prodrug

For a long time, CO has been considered a toxic substance. When it enters the blood, it reduces the oxygen-carrying capacity of hemoglobin, which can lead to permanent damage and even death [141]. However, recent studies have found that endogenous CO produced by heme oxygenase (HMOX) has a protective effect against tissue and cell damage [142, 143]. Endogenous CO is also a second messenger that regulates the cellular signaling pathway and participates in various physiological and pathological responses in the human body [141]. It has significant therapeutic potential in the treatment of many related diseases, including cerebral infarction, organ transplantation, arteriosclerosis, stroke and cancer [13, 144–148]. CO can promote the proliferation, metabolism and metastasis of tumor cells at low levels, while it can induce tumor cell death by interfering with mitochondrial respiration and increasing ROS at high levels, which lays a foundation for the treatment of tumors with CO [13, 144]. Therefore, the realization of a controlled-release CO prodrug is very important to improve the effectiveness of CO gas therapy and reduce the risk of CO poisoning. It is urgent to develop a nanogas delivery system that can target diseased tissues and control the release of CO gas [13].

### Photothermal therapy-triggered CO prodrug

**Fig. 4:**
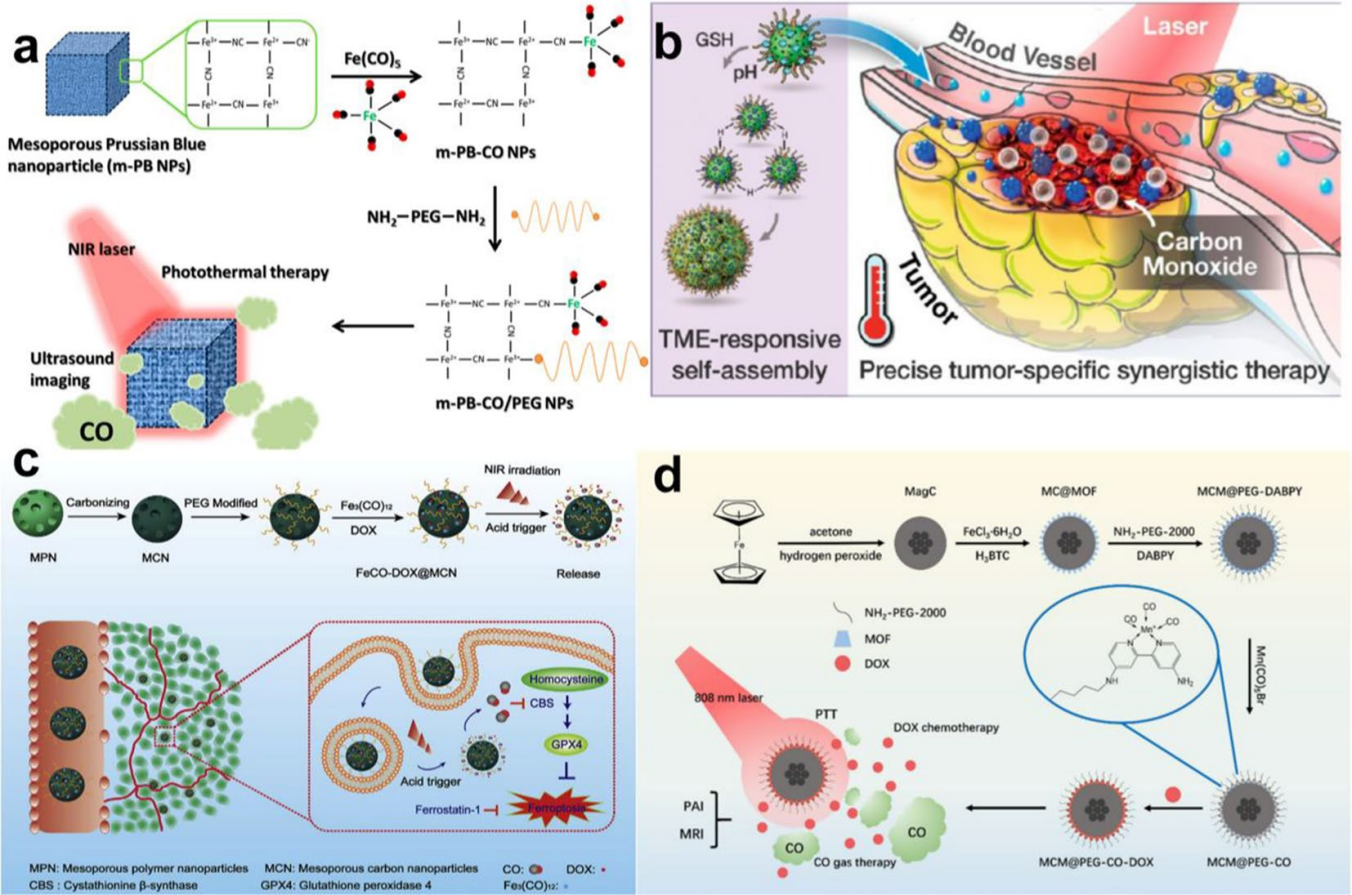
**a** Synthetic Strategy for NIR-Responsive m-PB-CO/PEG NPs Applied for CO and Photothermal Therapy and US Imaging. Reproduced with permission from Ref [87]. Copyright 2016, American Chemical Society. **b** Schematic Illustration of the Mn_2_(CO)10-Loaded and POM Surface-Modifified Hollow Mesoporous Organosilica Nanoplatform, HMOPM-CO, for Tumor Microenvironment (TME)-Responsive Self-Assembly and Precise Synergistic Therapy. Reproduced with permission from Ref [89]. Copyright 2018, American Chemical Society. **c** Schematic illustration of multifunctional nanoplatform for photoacoustic imaging-guided combined therapy enhanced by CO induced ferroptosis. Reproduced with permission from Ref [90]. Copyright 2019, Elsevier Ltd. **d** The synthetic workflflow of MCM@PEG-CO-DOX for NIR light-responded CO-DOX combination therapy of tumor with dual-mode imaging. Reproduced with permission from Ref [92]. Copyright 2019, Elsevier Ltd

To reduce the early leakage of CO in blood circulation and increase the accumulation of CO in tumor tissues, the use of NIR to stimulate CO prodrug nanoplatforms for on-demand release has attracted widespread attention in recent years. Prussian blue (PB) has been approved by the FDA in the United States as an antidote for heavy metal poisoning and has good biocompatibility and safety [149]. Therefore, Yeh et al. allowed polyethylene glycol carbonyl iron to react with mesoporous Prussian blue as the carrier to obtain a CO nanogas prodrug (m-PB-CO). Under 808 nm laser irradiation, the photothermal effect can trigger m-PB-CO to release CO. In contrast, in the absence of laser irradiation, m-PB-CO shows no CO release within 7 days, with high biological safety. In addition, m-PB-CO enables ultrasonic imaging under laser irradiation. In the mouse tumor model, the ultrasonic echo signal in the tumor was monitored by on/off pulsing of the laser. When m-PB-CO was injected into the tumor, compared with the group without laser irradiation, the ultrasonic signal of the tumor was significantly enhanced due to the release of CO after 5 min of 808 nm laser irradiation (Fig. 4a) [87]. Although PB nanoparticles have high biological safety, their photothermal conversion rate is not high. Palladium-loaded nanotablets (PdNS) have been widely used in NIR photothermal therapy because of their excellent photothermal conversion rate [150]. Wei et al. prepared ultrathin PdNS-CO nanocrystals using CO as a reducing agent. In addition, the photothermal conversion rate of PdNS-CO can be as high as 40%. After 808 nm laser irradiation, an excellent photothermal effect can trigger PdNS-CO to release CO, which can enrich PdNS-CO in tumors and enhance its antitumor effect by EPR [88].

Hollow mesoporous organosilicon nanoparticles (HMONs) have wide application prospects in the biomedical field because of their large specific surface area, uniform mesoporous structure, high chemical stability and controllable surface modification [151]. Chen et al. used a special “ammonia-assisted hot water etching” method to load Mo(VI)-based polyoxometalate (POM) and the CO-release molecule Mn_2_(CO)_10_ into HMONs. In the acidic tumor microenvironment, POM protonates and leads to the accumulation of HMONs in the tumor, enhancing the EPR effect of the tumor. In addition, the reductive tumor microenvironment will induce the reduction of Mo(VI) to Mo(V), which has stronger NIR light absorption. Therefore, under 808 nm irradiation, PTT can further trigger the thermal decomposition of Mn_2_(CO)_10_ and release CO, which acts synergistically with PTT (Fig. 4b) [89]. Compared with HMONs, mesoporous carbon nanoparticles (MCNs) have stronger absorbance, higher photothermal conversion efficiency and lower in vivo toxicity in the NIR region [152]. Yang et al. constructed an MCN nanoplatform (FeCO-DOX@MCN) loaded with DOX and triiron dodecacarbonyl (FeCO), which realized combined gas therapy and PTT. FeCO-DOX@MCN nanoparticles exhibit pH-dependent drug release behavior that can release more DOX in the acidic tumor microenvironment. Under 808 nm laser irradiation, the excellent photothermal conversion rate of FeCO-DOX@MCN can cause the thermal decomposition of FeCO to release CO. At the same time, CO can inhibit the expression of cystathionine β synthase (CBS) and glutathione peroxidase 4 (Gpx4), increasing the sensitivity of cancer cells to DOX by enhancing the effect of ferroptosis (Fig. 4c) [90].

As an excellent type of nanocarrier for drug delivery systems, metal organic frameworks (MOFs) have been widely used in a variety of drug carriers because of their low toxicity, high drug loading rate and high targeting [153]. Shen et al. formed MCM@PEG-CO-DOX nanoparticles by coloading DOX and CO prodrugs into an MOF and embedding polyethylene glycol magnetic carbon nanoparticles. Under 808 nm laser radiation, MCM@PEG-CO-DOX converts light energy into thermal energy, triggering CO and DOX release. In addition, MCM@PEG-CO-DOX can target the mitochondria of cancer cells to release CO, which can quickly cause mitochondrial damage and enhance the sensitivity of cancer cells to DOX, leading to cancer cell apoptosis. This will enhance the sensitivity of cancer cells to DOX, leading to apoptosis of cancer cells. In addition, MCM@PEG-CO-DOX can enable magnetic resonance imaging (MRI) and photoacoustic imaging (PAI) of tumors. Compared with non-intratumoral injection of the drug, intratumoral injection in mice resulted in T2 signal intensity at the tumor site of the mice with a lower signal area, while the PAI signal intensity showed a higher signal intensity [91]. Zhang et al. modified the surface of defective tungsten oxide (WO_3_) nanosheets (DW NSs) with bicarbonate (BC) by ferric ion-mediated coordination and further modified it with polyethylene glycol (PEG) to fabricate PEG@DW/BC nanosheets. Under 808 nm laser irradiation, PEG@DW/BC, producing a photothermal effect, can decompose BC to release CO_2_ and act as a CO photocatalyst to convert CO_2_ into CO. In addition, CO produced by PEG@DW/BC significantly inhibits the proinflammatory cytokines tumor necrosis factor-α (TNF-α) and interleukin-6 (IL-6), eliminating the inflammatory response produced by PTT (Fig. 4d) [92].

### Photodynamic therapy-triggered CO prodrug

ROS induced by NIR light not only can be activated by heat but also can trigger the release of CO [154]. The curative effect of PDT is mainly because the photosensitizer can produce a large amount of ROS under NIR light, which can induce the programmed death of cancer cells [155]. Although H_2_O_2_ produced by the PDT process was found to be involved in cancer cell apoptosis, long-term accumulation of high concentrations would lead to tumor recurrence and metastasis [156]. Therefore, Gu et al. reported a controlled CO-release system (CORM@G3DSP-CE6) driven by PDT that integrates the photosensitizer e6 chloride (Ce6) and the H_2_O_2_-sensitive CO-release molecule CORM-401 into a polypeptide dendrimer nanogel. Under laser irradiation, CORM@G3DSP-CE6 accumulates in the tumor, producing a large amount of H_2_O_2_, which could weaken the Mn-CO backbond in CORM-401 to release CO while being largely consumed. However, this process does not affect the production of ^1^O_2_, which not only retains the anticancer effect of PDT but also reduces the side effects of PDT. This will improve the anticancer effect [93]. There has been widespread interest in interference with cancer metabolism as an antitumor mechanism [157]. For example, starvation therapy can block tumor ATP supply by inhibiting the oxidative phosphorylation pathway, which can cause cancer cell necrosis [158]. Dong et al. designed a nanometallo-organic skeleton (NMOF) embedded with a photosensitizer (21-BODIPY) and CO prodrug (MnCO), which can achieve the synergistic effect of PDT and starvation therapy. Under NIR irradiation, the PDT process produces a large amount of ROS to promote the release of CO from MnCO. At the same time, PDT and CO gas therapy can cause mitochondrial damage and inhibit aerobic glycolysis, which blocks the energy supply of cancer cells, achieving effective combined treatment of cancer [94].

**Fig. 5:**
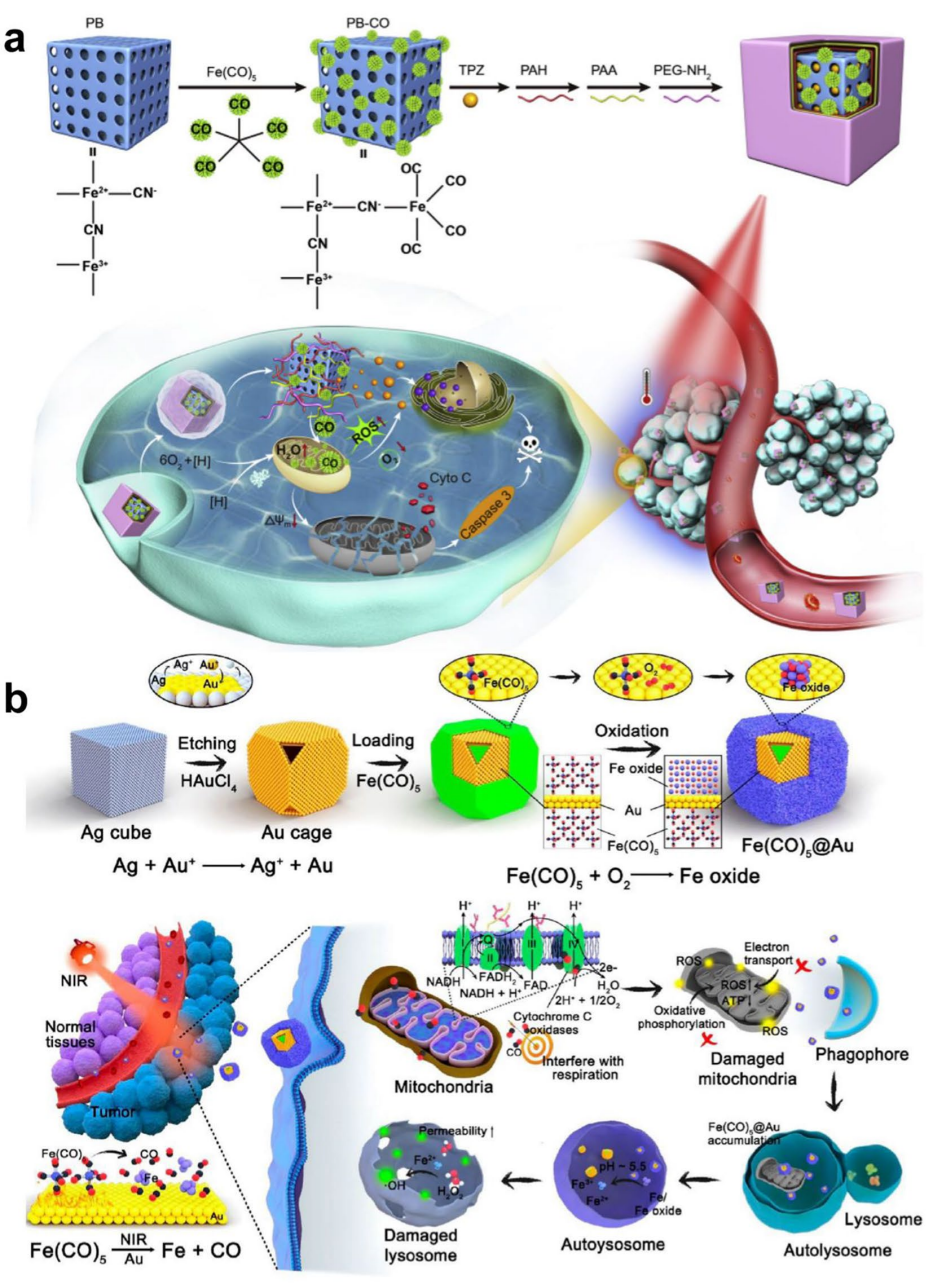
**a** Schematic illustration of PPPPB-CO-TPZ NPs with enhanced bioreductive chemotherapy and CO-mediated pro-apoptotic gas therapy. Reproduced with permission from Ref [95]. Copyright 2019, Elsevier Ltd. **b** Design of a controlled CO delivery nanomaterial for improving cancer therapy. Reproduced with permission from Ref [98]. Copyright 2020, American Chemical Society

Currently, some chemotherapeutic drugs can be fully activated in a specific tumor microenvironment, such as in the presence of overexpressed enzymes, overproduced ROS or hypoxia [159]. Yin et al. developed an NIR light-triggered CO release system, which consists of mesoporous Prussian blue nanoparticles (PB NPs) as a photosensitizer, pentacarbonyl iron (Fe(CO)_5_) as a CO donor and the bioreductive anticancer drug Tirapazamine (TPZ). Under 808 nm laser irradiation, the nanoparticles produce a large amount of ROS, leading to the decomposition of Fe(CO)_5_ and the release of a large amount of CO, which can cause mitochondrial damage and aggravate the hypoxic environment in the tumor by depolarizing the mitochondrial membrane. TPZ can be activated in the deep hypoxic tumor microenvironment, which aggravates the apoptosis of cancer cells and achieves a strong antitumor effect (Fig. 5a).[95] Currently, chemotherapy is still the main treatment for cancer in the clinic, but the MDR of cancer is a great obstacle to the efficacy of chemotherapy [131]. Yin et al. first used a CO nanodrug delivery system to overcome the MDR of tumors. Fe(CO)_5_ and DOX were coupled to mesoporous Prussian blue nanoparticles (PB NPs) to construct an NIR-responsive CO release system. Under 808 nm laser irradiation, the photothermal effect can cleave the Fe-CO bond to release CO, inducing mitochondrial damage. This process leads to the inhibition of APT-dependent drug efflux, which greatly increases the accumulation of DOX in tumor cells, overcoming the MDR of tumors. In addition, the large amount of ROS released during PDT can upregulate the expression of the proapoptotic protein caspase3 and induce apoptosis [96].

In recent years, nanocoordination polymers (NCPs) based on the combination of metal ions and organic compounds have attracted wide attention because of their high drug loading and high biosafety [160]. For example, NCPs containing copper ions have been used in antitumor therapy because of their excellent Fenton-like response in the tumor microenvironment [161]. Therefore, Wang et al. reported a GSH and NIR photoresponsive CO nano-prodrug composed of a CO donor (Fle) and Cu^2+^. The high concentration of GSH in the tumor microenvironment cleaves NCu-FleCP into smaller Fle and Cu^2+^, which enhances the drug uptake of cancer cells. In addition, high concentrations of H_2_O_2_ in the tumor microenvironment will react with Cu^2+^ to produce Fenton-like reactions to release a large amount of highly toxic·OH. Under irradiation with an 808 nm laser, the C–C bond of Fle is cleaved to release CO, causing damage to mitochondria. CO and·OH produced in situ can significantly promote the apoptosis of cancer cells, resulting in a significant synergistic anticancer effect [97]. Carbonyl compound complexes are easily oxidized under the physiological conditions of the human body, which inevitably leads to CO leakage [13]. Therefore, Zhang et al. encapsulated Fe(CO)_5_ in a Au nanocage cavity under anaerobic conditions and then formed iron oxide on the surface of Au nanocages under aerobic conditions, which effectively prevented CO leakage and oxidation, ensuring the stability and biocompatibility of the nanomaterials. After laser irradiation, Fe(CO)_5_ is thermally decomposed into CO and Fe. At this time, in the acidic tumor microenvironment, the iron oxide wrapped on the surface of the Au nanocage is decomposed by acid, releasing CO and Fe into the tumor in situ. CO gas can produce ROS by interfering with the mitochondrial respiratory chain, resulting in mitochondrial autophagy, which induces the accumulation of iron and iron oxide in lysosomes. The Fenton reaction of iron and iron oxide in acidic environments produces a large amount of hydrogen peroxide, which destroys lysosomes and accelerates the death of cancer cells (Fig. 5b) [98].

## NIR light-triggered H_2_S prodrug

In the past, hydrogen sulfide (H_2_S) was considered to be a highly toxic gas. In fact, it is also an endogenous cellular signal mediator that can transmit biological information between cells in physiology or pathology [11]. Therefore, endogenous H_2_S is considered to be the third gas transmitter in addition to NO and CO [10, 162, 163]. H_2_S signaling molecules show great potential in the treatment of many diseases, such as inflammation, diabetes and cancer [164–167]. It has been reported that a high concentration of H_2_S can produce a large amount of ROS, causing mitochondrial damage and inducing tumor cell apoptosis [167–169]. In recent years, the application of hydrogen sulfide in the medical field has aroused widespread interest, but controlling the release of H_2_S gas in time and space is still a difficult challenge. NIR-mediated PTT and PDT can stimulate H_2_S donors to release H_2_S, which has the unique advantages of being simple, noninvasive, safe and low in side effects [170–174]. Therefore, suitable photosensitizers are particularly important to control the release of H_2_S. At present, some photosensitizers have been used in the treatment of cancer, such as gold nanoparticles [175–180], carbon materials [181–183], copper sulfide nanoparticles [184–189], and organic dyes [190–195].

### Photothermal therapy-triggered H_2_S prodrug

**Fig. 6:**
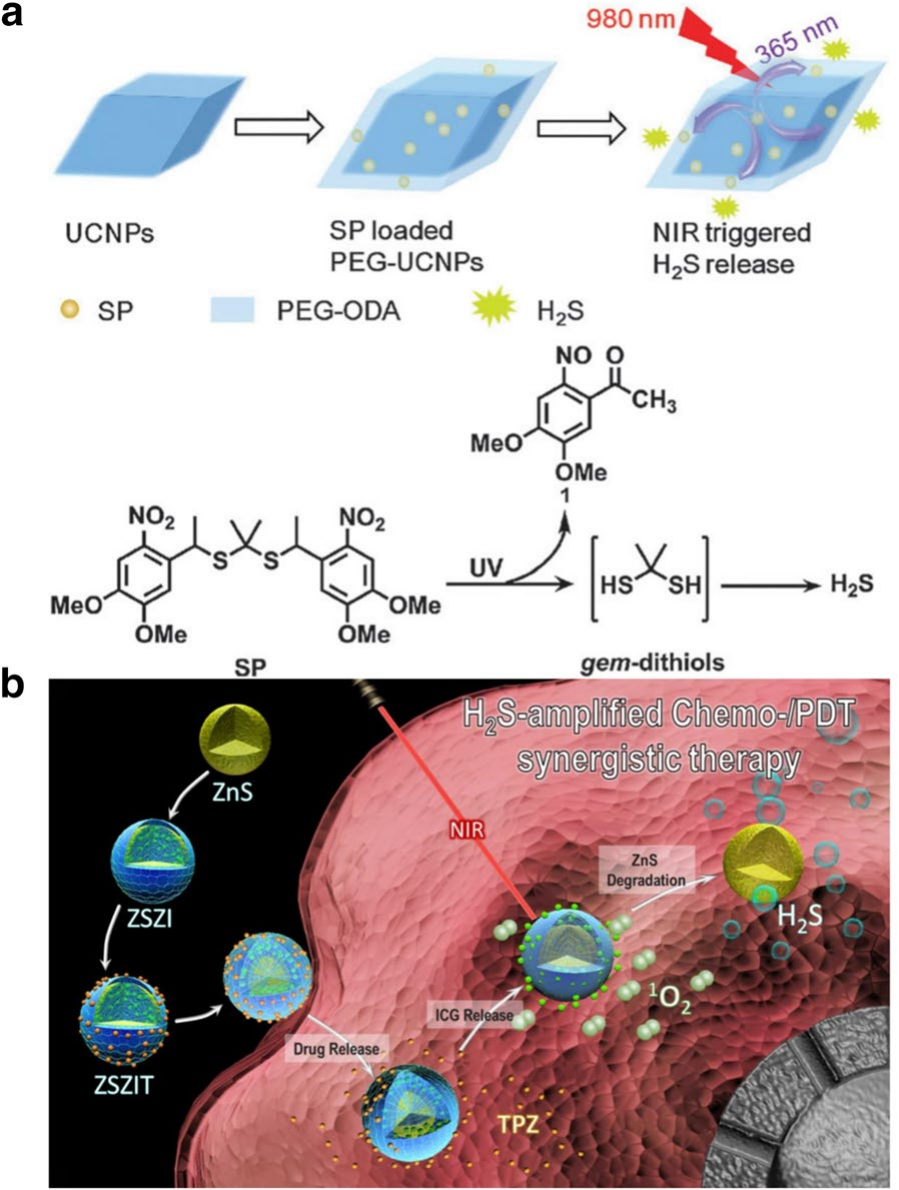
**a** Construction of SP-loaded PEG-UCNPs platform for NIR triggered H_2_S release. Reproduced with permission from Ref [99]. Copyright 2015, The Royal Society of Chemistry. **b** Schematic illustration of ZSZIT as a H_2_S-sensitized PDT/chemotherapeutic synergistic nanoplatform. Reproduced with permission from Ref [102]. Copyright 2020, Ivyspring International Publisher

In recent years, UCNPs have attracted widespread interest in the fields of biological imaging and antitumor activity because of their unique optical properties [167]. UCNPs can convert NIR light into UV or visible light, which enables their use in the biomedical field as carriers for the transmission or release of NIR light [196]. Liu et al. synthesized a new H_2_S donor (SP) for the first time and loaded it on the surface of UCNPs by hydrophobic interactions. Under 980 nm laser irradiation, the UV converted from NIR by UCNPs cleaves SP to gem-dithiols by luminescence resonance energy transfer (LRET). PTT can promote the thermal decomposition of gem-dithiols to release H_2_S gas. In addition, UCNPs can emit strong NIR fluorescence after NIR irradiation. SP-UCNPs were injected into mice and irradiated with 980 nm NIR light. The IVIS imaging system can track SP-UCNPs in vivo, which can regulate the targeted release of H_2_S (Fig. 6a) [99].

Reduced graphene oxide (rGO) has an excellent photothermal conversion rate for NIR and is a good carrier for photothermal therapy [197]. Dithiocarbamate (DTC) is the donor of H_2_S and can be pyrolyzed to H_2_S at high temperature [198]. Therefore, Liu et al. developed an NIR-photoresponsive H_2_S gas generation nanoplatform (rGO-PEI-DTC) with a high drug loading rate by the electrostatic adsorption of positively charged DTC and negatively charged rGO. Under irradiation with NIR light, light energy is converted into thermal energy based on the photothermal effect of rGO, and a large amount of H_2_S is released due to the thermal degradation of DTC. In the cell experiment, the rGO-PEI-DTC + NIR group showed higher cytotoxicity than the rGO and PEI-DTC groups, thus showing inhibition of the proliferation of cancer cells [100, 199].

Diallyl trisulfide (DATS), as a donor of H_2_S, can be decomposed into H_2_S by reductive GSH, which has good biological safety and strong cytotoxicity to cancer cells [200]. Zhang et al. constructed a complex gas-producing nanoplatform (Bi_2_S_3_-Ag_2_S-DATS@BSA-N_3_NYs) that responds to NIR light stimulation. In a tumor microenvironment rich in reductive GSH, DATS can be decomposed by GSH to release H_2_S. At the same time, the H_2_S released by the tumor in situ can reduce the surface –N_3_ (−) to –NH_2_ ( +), resulting in the adsorption of negatively charged BSA and increasing the size of NYs, which effectively enhances the enrichment of nanodrugs in the tumor site. Under 808 nm laser irradiation, the photothermal conversion rate of the nanoparticles was as high as 31.6%, which indicates an excellent photothermal effect. In addition, Bi_2_S_3_-Ag_2_S-DATS@BSA-N_3_NYs were injected into mice through the tail vein, and NIR-II fluorescence and PA imaging were performed. After 6 h, the NIR-II fluorescence and PA signal of the tumor were significantly higher than those of the surrounding normal tissue. Therefore, NIR-II/PA dual-mode imaging-guided PTT and GT provides a promising method for effective antitumor therapy [101].

### Photodynamic therapy-triggered H_2_S prodrug

PDT can stimulate H_2_S gas for use in antitumor therapy. TPZ is a hypoxia-activated anticancer drug that is highly toxic to cancer cells in the hypoxic tumor microenvironment but has little effect on cells with normal oxygen concentrations [201]. High expression of catalase (CAT), which is overexpressed in the tumor microenvironment, can convert H_2_O_2_ into oxygen, relieving hypoxia and weaking the anticancer effect of TPZ [202]. However, H_2_S can inhibit the expression of CAT [203]. Han and others have developed a H_2_S nanogas production platform (ZSZIT) composed of ZnS nanoparticles coated with zeolitic imidazolate framework-8 (ZIF-8) and combined with indocyanine green (ICG) and TPZ. In the acidic tumor microenvironment, the shell of ZIF-8 collapses due to protonation, releasing ICG and TPZ. Under NIR radiation at 808 nm, ICG can induce PDT to produce ROS, which consume a large amount of oxygen at the tumor site, aggravating hypoxia in the tumor microenvironment. More importantly, ZnS can be degraded in situ to form H_2_S gas in the tumor. H_2_S can not only kill cancer cells but also downregulate the expression of CAT. This process aggravates the anoxic environment of the tumor tissue by blocking the transformation of H_2_O_2_ to O_2_ and activating and enhancing the cytotoxicity of TPZ. Therefore, due to the synergistic effects of PDT, H_2_S and TPZ, ZSZIT has obvious antitumor effects in vivo and in vitro, indicating great potential in cancer treatment (Fig. 6b) [102].

## NIR light-triggered H_2_ prodrug

Gas therapy, a relatively new treatment method, mainly uses gas molecules (NO, CO, H_2_S, H_2_ and SO_2_) to treat various diseases [204, 205]. Among them, H_2_ has higher biosafety than other gases, which has attracted increasing attention. In 1975, Dole et al. found that high concentrations of H_2_ can be used to treat skin cancer [206]. In 2007, H_2_ was proven to be able to scavenge harmful free radicals such as hydroxyl (·OH) and peroxynitrite (ONOO^−^), thereby reducing inflammation or ischemia–reperfusion damage [14]. Since then, a series of studies have shown that H_2_ has a significant therapeutic effect on a variety of diseases, including cancer, diabetes and neurodegeneration [14, 207, 208]. Unlike other therapeutic gas transmitters, hydrogen has no risk of poisoning even at high concentrations. However, the solubility of hydrogen is low, and it can diffuse arbitrarily in the body; as a result, directly inhaling hydrogen or injecting/drinking hydrogen-rich water is not an easy method to reach and accumulate a large number of hydrogen molecules in deep lesions [209]. Therefore, how to achieve hydrogen targeting and controllable and continuous hydrogen release at the target site through nanosystems is the main challenge at present.

### Photothermal therapy-triggered H_2_ prodrug

Hydrogen is a flammable and explosive gas but has been regarded as biologically inert for a long time. However, a large number of recent studies have shown that in the physiological environment, hydrogen is an endogenous signaling molecule with good biosafety and is considered to be a reductive homeostatic regulator [210]. It has shown certain effects on many diseases related to inflammation and oxidation, such as cancer, ischemia–reperfusion injury, and cardiovascular disease. [209, 211, 212] He et al. put forward the concept of “hydrothermotherapy” for the first time, using small palladium nanoparticles as hydrogen carriers and self-catalysts to form stable PdH 0.2 nanoparticles, realizing tumor delivery by passive targeting and achieving the efficacious PTT and photoacoustic imaging [213]. Palladium hydride nanomaterials (PdH 0.2) are used for targeted hydrogen delivery and controlled release in tumors to achieve efficient hydrogen thermotherapy. However, the hydrogen carrying capacity of the synthesized PdH 0.2 is limited (H:Pd = 0.2). The synthesis of palladium hydride with a high hydrogen carrying capacity needs to be carried out under high pressure, and the stability of the synthesized product is poor. Recently, to increase the hydrogen loading capacity, a research group proposed using palladium as the coordination center and tetrapyridyl porphyrin as the ligand to synthesize a new type of PdH-MOF nanomaterial in one step. With the help of monatomic palladium in the MOF construction unit, the efficient loading of hydrogen (H:Pd = 1) and long-term slow release of hydrogen were realized. In addition, hydrogen loaded with monatomic palladium has high catalytic activity as a highly reductive form of hydrogen, which is beneficial for the scavenging of highly oxidizing ROS. The photothermal conversion efficiency of PdH-MOF is up to 44.2%, providing a good photoacoustic imaging effect. Combined with the self-fluorescence characteristics of porphyrin, it can be used for in vivo tracking and treatment guidance of nanoparticles and can also be combined with photothermal effects to achieve hydrogen-thermal combined antitumor therapy [103].

**Fig. 7:**
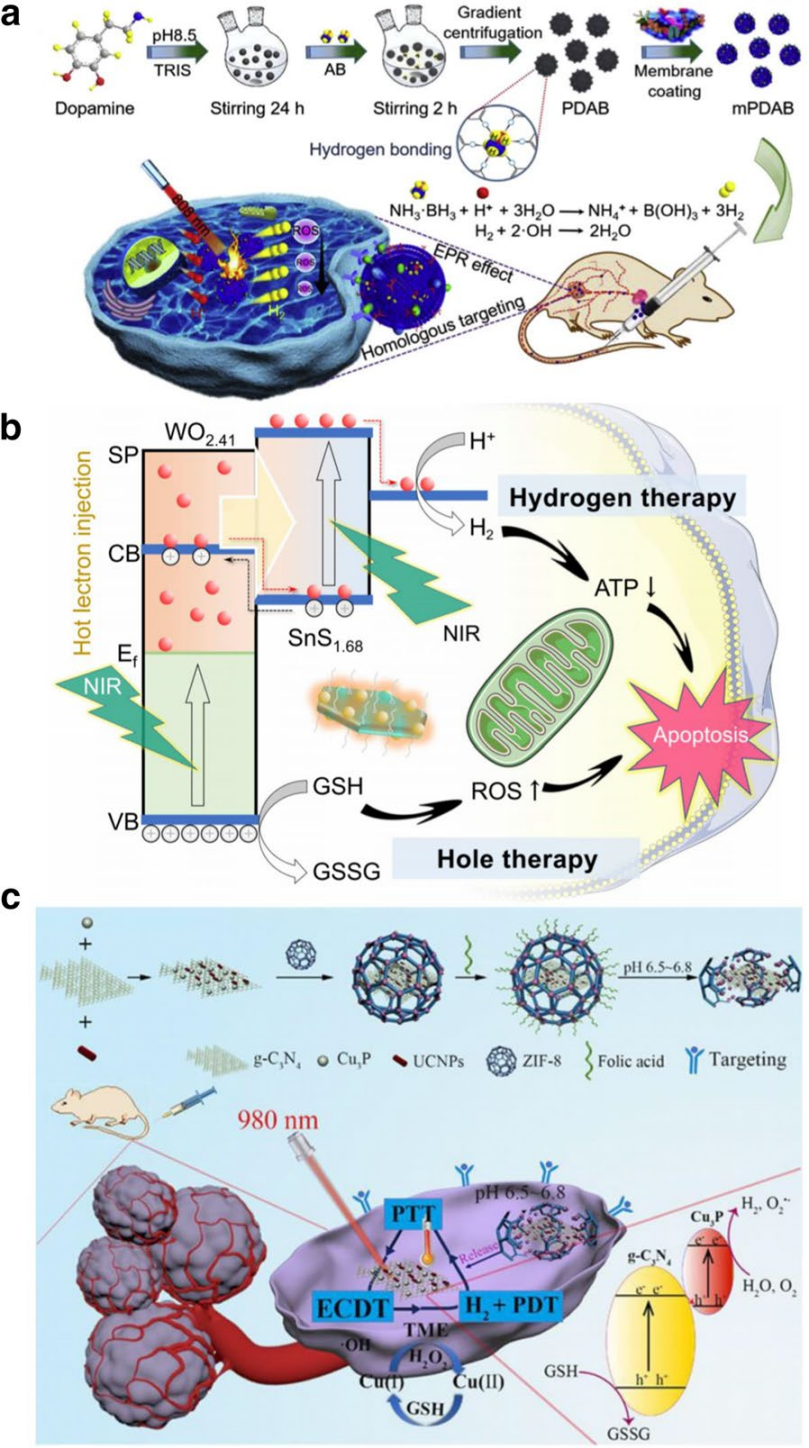
**a** Schematic illustration of mPDAB for tumor therapy. Reproduced with permission from Ref [104] Copyright 2019, Elsevier Ltd. **b** Schematic illustration of combined hole/hydrogen therapy strategy and mechanisms with the NIR-activable Z-scheme SnS_1.68_–WO_2.41_ Nanocatalyst. Reproduced with permission from Ref [105]. Copyright 2021, Nature Publishing Group. **c** Schematic Illustration of the Multimodal Nanoplatform for H_2_-Mediated Cascade-Amplifying Synergetic Therapy. Reproduced with permission from Ref [106]. Copyright 2020, American Chemical Society

Although PTT can kill cancer cells at high temperature, this process is often prone to inflammatory reactions, leading to tumor recurrence, metastasis and other adverse consequences [214]. For this reason, Zhang et al. constructed a biofilm camouflage nanodrug (mPDAB) containing PDA and aminoborane (AB). In the acidic tumor microenvironment, AB releases H_2_ and reacts with ·OH in situ, which reduces the inflammatory response induced by PTT by inhibiting the increase in intracellular ROS induced by PTT and downregulating the levels of TNF-α and IL-6. In addition, after labeling mPDAB with Cy5.5, the biological distribution of nanodrugs in vivo was studied by using a small-animal fluorescence imaging system. The results showed that the fluorescence intensity of Cy5.5 in the tumor site was significantly enhanced over time, indicating that the accumulation of mPDAB in the tumor site was stronger. This is due to biofilm recombination, which gives mPDAB a longer blood circulation time and enables homologous targeting in vivo. In the mouse tumor model, the distant metastasis of tumors was greatly inhibited in the mPDAB + NIR group because the expression of the tumor proliferation marker Ki67 was significantly decreased (Fig. 7a) [104].

Recently, a Z-type SnS1.68-WO2.41 nanocatalyst was constructed by riveting WO2.41 nanodots on the surface of SnS1.68 nanowires (1.49 eV). WO2.41 nanoparticles injected hot electrons into SnS1.68 nanowires through the surface plasma effect to achieve NIR photocatalytic hydrogen production while enhancing the oxidation ability of the system so that the overexpressed GSH in tumors could be used as a reducer to realize NIR photocatalytic hydrogen production in tumors. The increase in hydrogen and the deprivation of GSH synergistically inhibit the proliferation of cancer cells and induce their apoptosis, leading to the degeneration of overgrown tumor vessels and greatly reducing the content of tumor-associated macrophages (removal of tumor immunosuppression), thus effectively destroying the tumor microenvironment in which cancer cells survive (Fig. 7b) [105].

### Photodynamic therapy-triggered H_2_ prodrug

H_2_ can affect the level of ROS in tumor cells through NIR and induce cancer cell injury and apoptosis [215]. However, the low solubility of H_2_ in water makes it spread easily in the blood and prevents enrichment in the tumor site [209]. It is well known that the water content of the human body ais approximately 70%, which provides us with a way of thinking about how to introduce photocatalytic nanomaterials into tumors and suggests that catalyzing water to produce H_2_ will be a promising strategy to enhance H_2_ accumulation in tumors [216]. In recent years, Z-scheme heterojunction systems have been widely used in the generation of PDT and H_2_ because they can separate electron–hole pairs and improve redox potential [217]. Wang et al. constructed a NIR-photoresponsive in situ hydrolytic hydrogen production nanoplatform (UCCZ-FA) that uses ZIF-8 as its shell and introduces a gC_3_N_4_/Cu_3_P Z-scheme heterojunction for light-induced ROS and H_2_ production. Under the guidance of folic acid, when UCCZ nanoparticles were actively targeted to enrich the tumor, the acidic tumor microenvironment caused the ZIF-8 shell to collapse and release UCC composite nanoparticles. Under 980 nm laser irradiation, the electrons produced by gC_3_N_4_ recombine with the electron holes of the Z-scheme, while the high concentration of GSH in the tumor microenvironment can inhibit this process and transfer electrons to Cu3P to catalyze H_2_O to produce H_2_ and O_2_^−^. In addition, CU (I) reduced H_2_O_2_ to highly toxic ·OH by the Fenton reaction. More importantly, H_2_ can inhibit the inflammation caused by oxidative stress and PTT in PDT, which promotes the apoptosis of cancer cells by upregulating the expression of Caspase-3 protein. This combination of GT, PTT, PDT and CDT can effectively inhibit tumor growth (Fig. 7c) [106].

## NIR light-triggered SO_2_ prodrug

Sulfur dioxide (SO_2_) has always been considered an air pollutant. In fact, it is also a therapeutic gas transmitter, and can cause oxidative damage to tumor cells by exhausting glutathione in the tumor microenvironment and destroying the membrane structure [218, 219]. According to recent studies, SO_2_ has great therapeutic potential in a variety of diseases, including cardiovascular disease, inflammation and cancer [220–223]. However, the biological toxicity and low stability of SO_2_ gas limit its clinical application in vivo. In this context, it is urgent to develop a gas-producing nanosystem for the targeted transport and controlled release of SO_2_ gas. To date, small molecular prodrugs based on different endogenous and exogenous stimulus release mechanisms have been developed, including GSH, pH and light-induced release [224–226]. Among them, NIR more easily achieves the on-demand release of SO_2_ in deep tumor tissues, which can prevent phototoxicity caused by ultraviolet light.

### Photothermal therapy-triggered SO_2_ prodrug

MDR of cancer cells is one of the main obstacles hindering the effect of cancer chemotherapy [227]. A high concentration of GSH in the tumor microenvironment is one of the important causes of tumor MDR, which protects cancer cells from ROS and maintains tumor redox homeostasis [228]. According to studies, reducing the concentration of GSH can increase the efficacy of anticancer drugs to overcome the MDR of tumor cells [229]. Chen et al. made great progress in the fight against tumor MDR by triggering the release of SO_2_ gas and DOX by glutathione [224]. However, their research methods cannot control and monitor the release of SO_2_ and DOX in real time. Therefore, N.D. PradeepSingh et al. designed a SO_2_ gas drug delivery system (DDS) that overcomes the MDR of tumors by loading the anticancer drug chlorambucil and using GSH and NIR as stimulation conditions. In the tumor microenvironment, DDS reacts with a high concentration of GSH to produce SO_2_ gas with green fluorescence, which helps to better distinguish cancer cells from normal cells. Under irradiation with NIR light, when the anticancer drug chlorambucil is released at the tumor site, blue fluorescence can be emitted. In addition, the released SO_2_ can reduce the concentration of GSH and enhance the efficacy of chlorambucil, overcoming the MDR of tumors. This dual stimulus gas delivery system monitors SO_2_ gas release and chlorambucil in real time under two kinds of fluorescence, which not only enhances the anticancer efficacy but also ensures the biosafety of drugs [107].

### Photodynamic therapy-triggered SO_2_ prodrug

**Fig. 8:**
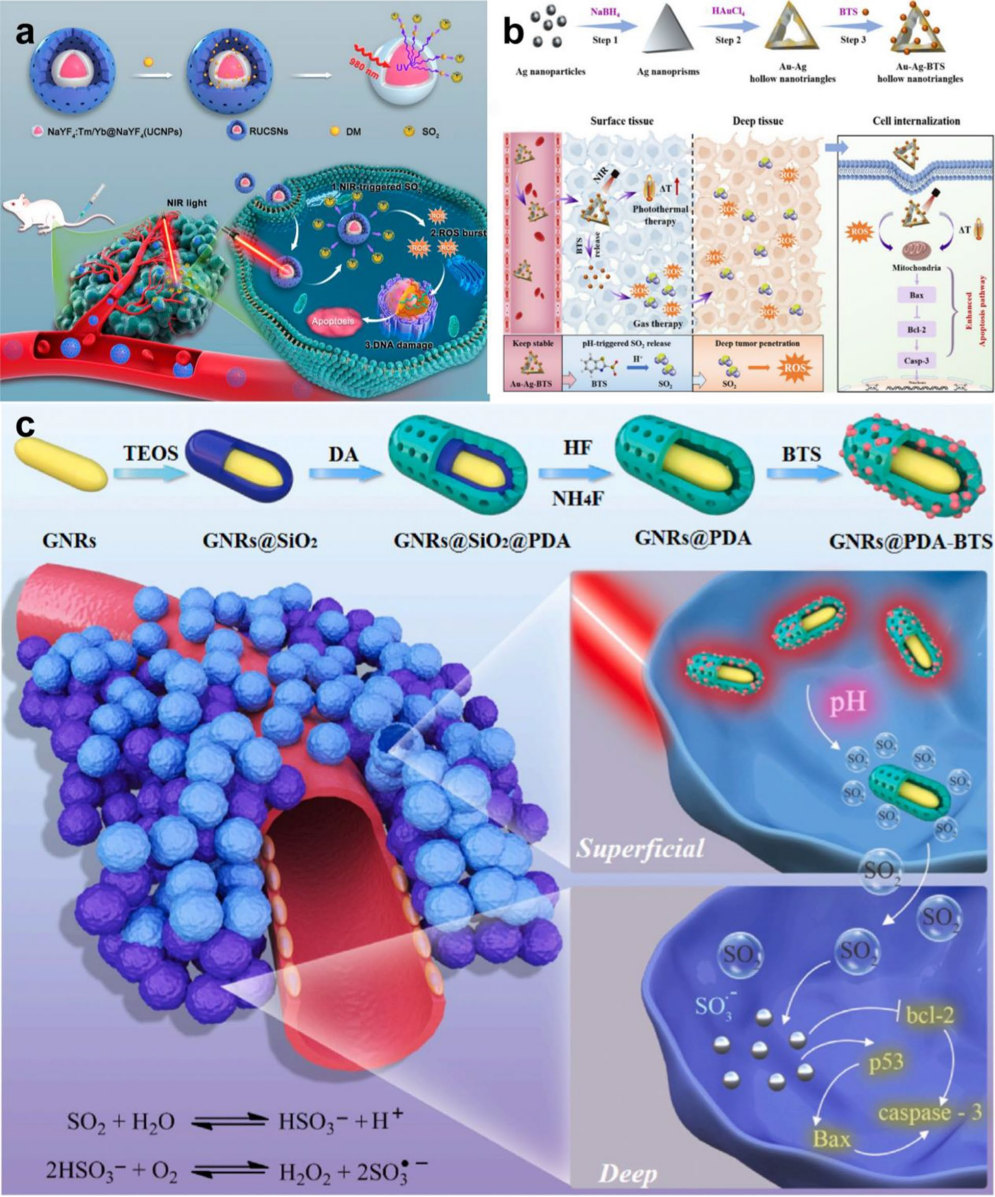
**a** Intracellular localized SO_2_ generation and therapeutic action upon NIR light irradiation after cell uptake of RUCSNs-DM. Reproduced with permission from Ref [108]. Copyright 2019, American Chemical Society. **b** Schematic illustration of synthetic procedure of Au-Ag-BTS HTNs, and a win–win therapeutic mechanism of Au-Ag-BTS HTNs by acting together on apoptosis protein—Bax and enhanced tumor penetration. Reproduced with permission from Ref [109]. Copyright 2020, Elsevier Ltd. **c** Schematic illustration of the preparation of GNRs@PDA-BTS and the design of GPBRs for gas therapy and photothermal therapy. Reproduced with permission from Ref [110]. Copyright 2020, Elsevier Ltd

SO_2_ can increase the level of ROS, lead to DNA damage, and finally induce apoptosis of cancer cells [230].Yang et al. developed an NIR-photoresponsive SO_2_ gas nanoplatform (RUCSNs-DM) based on hollow mesoporous silica-embedded UCNPs and SO_2_ donors (the 1-(2,5-dimethylthien-1,1-dioxide-3-yl)-2-(2,5-dimethylthien-3-yl)-hexaflfluorocyclopentene, DM). UCNPs can convert NIR light into ultraviolet light, which leads to the breaking of C-S bonds in DM and the controlled release of SO_2_. RUCSNs-DM showed good biological safety, and the cytotoxicity to cancer cells without NIR irradiation was negligible. In addition, the SO_2_ produced by RUCSNs-DM in cancer cells greatly increased the concentration of ROS, resulting in apoptosis and DNA damage. In the mouse tumor model, the antitumor effect of RUCSNs-DM combined with NIR was significantly better than that of the control groups. The 30-day survival rate of mice in this group reached 100%, while showing very low side effects (Fig. 8a) [108].

At present, a large number of precious metal nanoparticles are widely used in antitumor research, among which Au–Ag alloys are widely used in photothermal agents because of their excellent photothermal effect [231]. Li et al. constructed a novel SO_2_ gas generation nanoplatform based on Au–Ag high-temperature superconducting nanotubes (Au–Ag HTNS) as carriers and combined it with the SO_2_ donor benzothiazole sulfinate (BTS). Au-Ag-BTS has excellent heat conversion ability and does not produce thermal attenuation after many experiments, so it can be used as an effective photosensitizer for accurate tumor phototherapy. Under acidic condition, the C-S bond of BTS was destroyed and released SO2 that combined with PTT up-regulated the expression of Bax and Caspase-3 and inhibited the expression of Bcl-2, which significantly promoted the apoptosis of cancer cells. According to the fluorescence localization imaging, Au-Ag-BTS HTNS can also be internalized and released SO2 by lysosome pathway, resulting in the increase of ROS. This method of synergistic elimination of deep tumors by PTT and GT has achieved excellent anti-tumor efficacy. In addition, Au-Ag-BTS HTNS can also be used as a contrast agent for computed tomography (CT) to better guide the antitumor therapy of PTT and PDT. The nanosystem can effectively solve problems related to the biocompatibility, targeting, and intracellular and in vivo targeted delivery of SO_2_ (Fig. 8b) [109].

In addition, the group constructed gold nanorods@mesoporous dopamine (GNRS@PDA-BTS) based on BTS. Because of the large amount of amino groups in PDA, the drug loading rate of the nanosystem SO_2_ is as high as 80%. In an acidic environment, the Cmure S bond in BTS breaks and releases SO_2_. PTT can then promote the continuous release of SO_2_. In addition, SO_2_ can enhance the efficacy of PDT by increasing the concentration of ROS, upregulating the expression of the proapoptotic proteins p53, bax, and caspase-3, and downregulating the expression of the antiapoptotic protein bcl-2 to effectively promote the apoptosis of cancer cells (Fig. 8c) [110].

## Conclusions and outlook

In summary, gas therapy is a new and promising anticancer therapy strategy. In recent years, the introduction of nanotechnology into the construction of nanogas prodrugs has greatly promoted the development of precision gas nanomedicine in the field of biomedicine. NO, CO, H_2_, H_2_S and SO_2_ at appropriate concentrations have excellent antitumor effects with low systemic side effects. NIR light mainly triggers the breaking of chemical bonds in nano-prodrugs by PTT and PDT to release gas molecules on demand, which is highly controllable. Although great progress has been made in gas therapy, most gas molecules are prone to nonspecific distribution after systemic administration, resulting in strong toxicity to normal tissues. Therefore, developing nanogas prodrugs with targeted transport and controlled release is the main problem before clinical transformation. As a specific noninvasive stimulus with a high penetration depth, NIR light has been widely used in PTT/PDT-triggered gas release from nano-prodrugs. This paper introduces in detail the latest progress in the application of NIR photoresponsive nanogas prodrugs in the field of antitumor therapy in recent years, including how to make use of the unique tumor microenvironment (such as pH, GSH, H_2_O_2_ and ATP) to achieve the precise release of gas molecules in the tumor site. Although gas therapy has achieved exciting results so far, it still faces challenges that urgently need to be solved.

First, although therapeutic gas molecules show good biosafety and low toxicity at appropriate concentrations due to the controlled release of NIR light-responsive nanocarriers, nanocarriers will still have some unpredictable biosafety problems. For example, in some special physiological environments of organisms, nanocarriers show a high degree of instability, which will lead to the premature release of gas molecules. In addition, although inorganic nanomaterials show a high degree of stability, they may pose a threat to the life of organisms as endogenous toxicants. Therefore, the combination of organic–inorganic nanomaterials may be able to overcome the shortcomings of both and while maintaining good biosafety at the same time. However, before the realization of clinical transformation, long-term systematic studies of their biosafety are still needed, including pharmacodynamics, pharmacokinetics and biodegradability. Second, although a variety of NIR light-responsive gas prodrugs have been developed, the progress of gas therapy has been greatly promoted. However, these gas molecules and nanocarriers are rarely used in clinical trials. Some promising nanocarriers have excellent versatility, but the complex preparation process greatly limits the possibility of clinical transformation. In clinical transformation, improving the repeatability, stability and efficiency of nanocarrier preparation is a key step to achieve clinical transformation.

Therefore, as a popular cutting-edge science, gas therapy can not only exert the therapeutic function of gas itself but can also be combined with gas therapy to increase the antitumor effect. Although gas therapy is currently still in its infancy, clinical conversion will benefit patients, which will require the joint efforts and contributions of researchers and experts from various industries.

## Data Availability

Not applicable.
